# Restoring Anterior Guidance With a Customized Incisal Guide Table Using the Admix Technique: A Case Report

**DOI:** 10.7759/cureus.116534

**Published:** 2026-09-19

**Authors:** Meenakshi Thimmappa, Priya Shaw, Bhoomika B M, Prateeka Veerapur

**Affiliations:** 1 Prosthodontics and Crown and Bridge, The Oxford Dental College, Bengaluru, IND

**Keywords:** admix technique, anterior guidance, customized incisal guide table, dental occlusion, fixed prosthodontics, posterior disocclusion

## Abstract

Anterior guidance is a key determinant of functional occlusion, directing mandibular movements and facilitating posterior disocclusion during eccentric excursions. Accurate reproduction of anterior guidance during prosthodontic rehabilitation is essential for achieving occlusal harmony, minimizing interferences, and enhancing the longevity of restorations. Conventional articulator settings may not adequately replicate patient-specific mandibular movements, making customization of the incisal guide table a valuable adjunct in treatment. This case report describes the restoration of anterior guidance in a patient with missing mandibular anterior teeth using a customized incisal guide table fabricated by the admix technique. Diagnostic casts were mounted on a semi-adjustable articulator, and a diagnostic wax-up was performed to establish the proposed occlusal scheme. Functional protrusive and lateral mandibular movements were recorded intraorally and transferred to the articulator through customization of the incisal guide table using admix material. The customized guide table enabled accurate reproduction of functional mandibular pathways during fabrication of the definitive fixed prosthesis, facilitating the establishment of harmonious anterior guidance and a mutually protected occlusal scheme. Follow-up evaluation demonstrated satisfactory esthetic and functional outcomes, improved masticatory efficiency, stable occlusion, and absence of complications. This technique offers a simple, economical, and clinically reliable method for accurately transferring functional mandibular movements and achieving predictable occlusal rehabilitation.

## Introduction

Anterior guidance is an essential component of functional occlusion, directing protrusive and lateral mandibular movements while protecting posterior teeth from excessive horizontal and oblique forces. Proper anterior guidance facilitates posterior disocclusion during eccentric movements, thereby reducing occlusal interferences, minimizing wear, and contributing to the longevity of both natural dentition and prosthetic restorations [1-3].

Loss of anterior guidance may occur as a result of trauma, attrition, periodontal disease, or extraction of anterior teeth, leading to altered occlusal dynamics, compromised mastication, and increased stress on the stomatognathic system. In fixed prosthodontic rehabilitation, restoration of anterior guidance is critical not only for replacement of missing teeth but also for re-establishing functional occlusion and biomechanical stability [2,3]. However, reproducing patient-specific mandibular movements during prosthesis fabrication remains a clinical challenge, as conventional articulator settings may not adequately duplicate individualized functional pathways [4].

Customization of the incisal guide table enables the recording and transfer of protrusive and lateral mandibular movements to the articulator, facilitating accurate reproduction of anterior guidance during prosthesis fabrication. Although various materials have been used for this purpose, the admix technique offers a simple, economical, and easily modifiable method for recording functional mandibular movements [5]. This case report describes the rehabilitation of a patient with missing mandibular anterior teeth using a customized incisal guide table fabricated with the admix technique to restore anterior guidance and establish harmonious occlusion.

The present case report has been reported following the CARE (Case REport) guidelines for case reports [6].

## Case presentation

Patient information

A 42-year-old female patient presented to the Department of Prosthodontics and Crown & Bridge with the chief complaint of missing lower front teeth and difficulty chewing. The patient expressed concern regarding compromised esthetics and requested a fixed replacement. Medical, family, and psychosocial histories were non-contributory. The patient was cooperative and categorized as a philosophic-type patient. The mandibular anterior teeth (31, 32, 41, and 42) had been extracted three months earlier due to periodontal disease.

Clinical findings

Extraoral examination revealed no facial asymmetry, and temporomandibular joint movements were within normal limits. Intraoral examination demonstrated an edentulous span in relation to 31, 32, 41, and 42 (Figure 1), with adequate restorative space and a favorable residual ridge morphology. Clinical examination revealed loss of anterior guidance and compromised esthetics.

**Figure 1:**
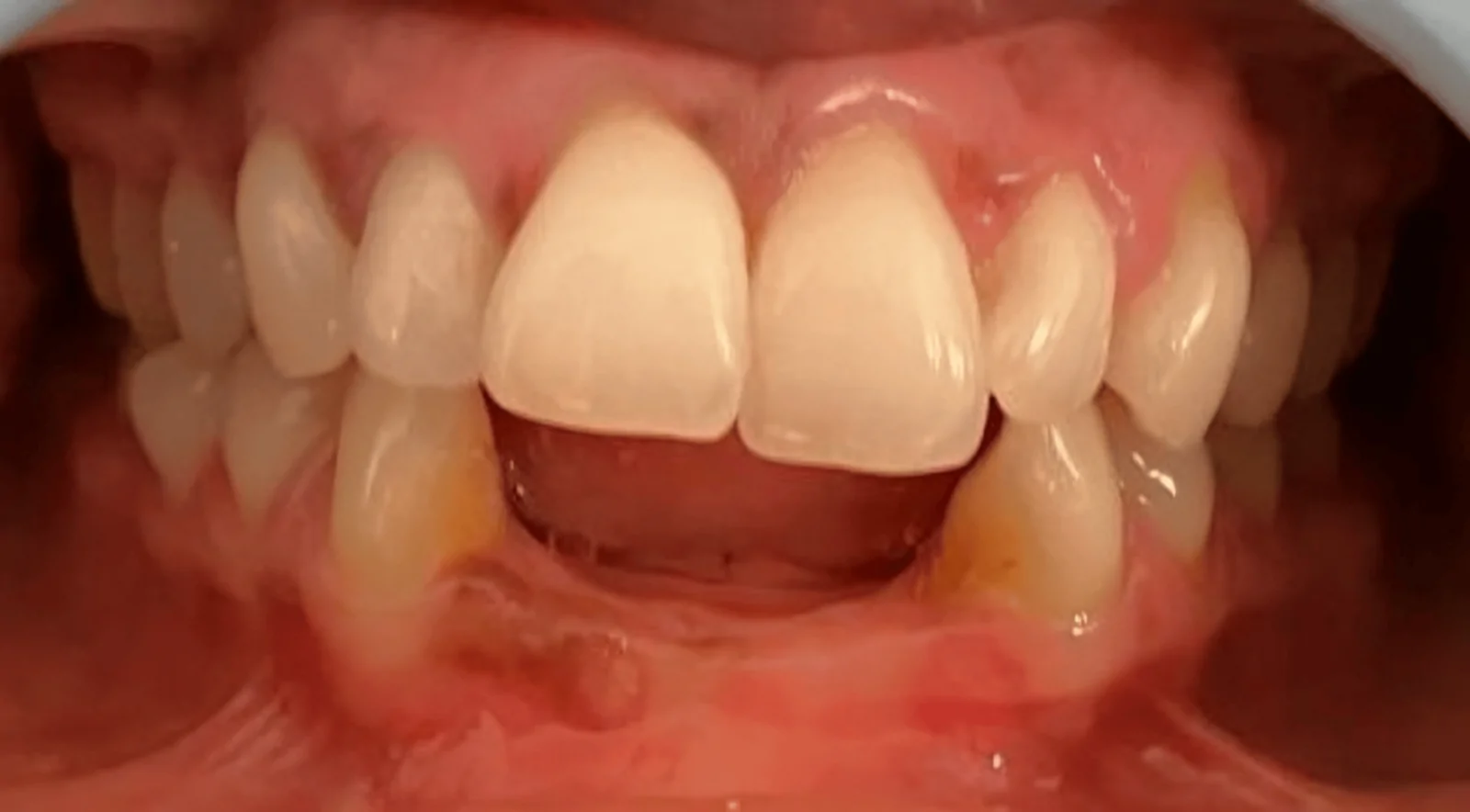
Intraoral view showing missing 31, 32, 41, and 42

Cone beam computed tomography (CBCT) (Figure 2) confirmed the absence of the mandibular incisors and revealed favorable conditions for prosthodontic rehabilitation. Based on the clinical and radiographic findings, the case was classified as a moderately compromised condition according to the American College of Prosthodontists (ACP) classification [7].

**Figure 2:**
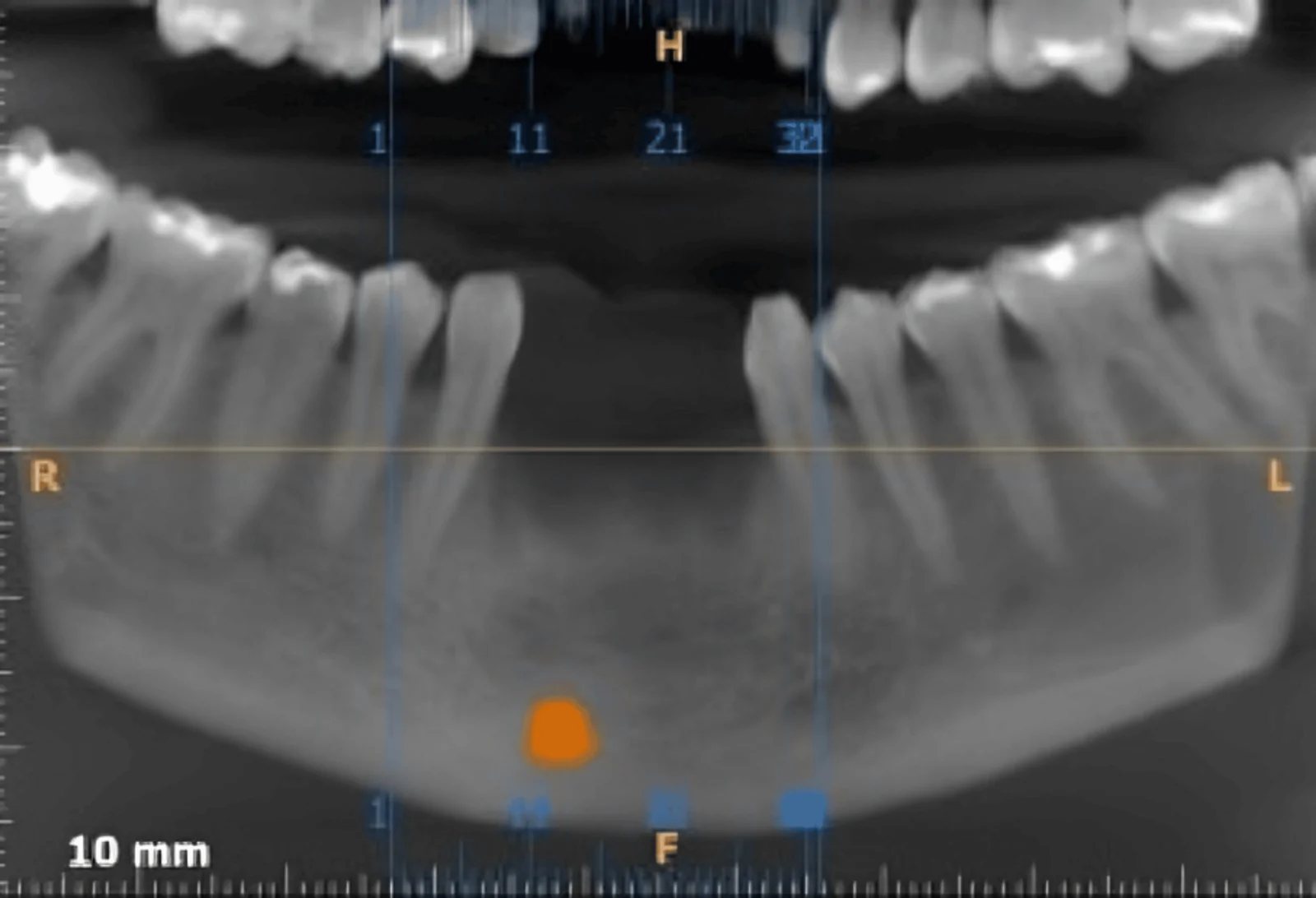
Cone-beam computed tomography image demonstrating the absence of the mandibular incisors

Table 1 presents the chronological timeline of the patient’s clinical course.

**Table 1:** Timeline of the patient's clinical course

Time	Clinical event
3 months prior to presentation	Extraction of teeth 31, 32, 41, and 42 due to periodontal compromise
Initial visit	Clinical examination, patient history, and radiographic assessment
Diagnostic phase	Diagnostic impressions, cast fabrication, facebow transfer, mounting on semi-adjustable articulator, occlusal analysis, and fabrication of customized incisal guide table
Provisional restoration phase	Tooth preparation and fabrication of provisional fixed dental prosthesis; evaluation of esthetics, phonetics, comfort, and anterior guidance
Definitive treatment	Final impressions, fabrication, and cementation of all-ceramic fixed dental prosthesis
6-month follow-up	Clinical and radiographic evaluation

Diagnostic assessment

Clinical examination, CBCT imaging, diagnostic casts, and occlusal analysis were used to establish the diagnosis and formulate the treatment plan. The residual ridge corresponded to a Seibert Class I defect. The patient was diagnosed with partial edentulism involving missing teeth 31, 32, 41, and 42, resulting in loss of anterior guidance and altered occlusal dynamics.

Treatment options, such as a removable partial denture, tooth-supported fixed prosthesis, and implant-supported prosthesis, were discussed with the patient. As per the patient's choice, tooth-supported fixed prosthetic rehabilitation was selected to restore esthetics, function, and anterior guidance.

Diagnostic challenges

No significant diagnostic challenges were encountered. However, accurate reproduction of patient-specific mandibular movements and restoration of anterior guidance represented a clinical challenge during prosthodontic rehabilitation.

Prognostic characteristics

The prognosis was considered favorable because of adequate periodontal support of the abutment teeth, favorable residual ridge morphology, sufficient restorative space, absence of systemic risk factors, and patient compliance with oral hygiene instructions.

Therapeutic intervention

Type of Therapeutic Intervention

A fixed prosthodontic rehabilitation was planned to restore the missing mandibular anterior teeth and re-establish anterior guidance using a tooth-supported fixed partial denture. Teeth 33, 34, 43, and 44 were selected as double abutments based on their favorable periodontal and clinical status, including adequate periodontal support, absence of mobility, positive vitality responses, and satisfactory root morphology and alveolar bone support on radiographic examination, with no evidence of periapical pathology. The use of double abutments on each side increased the overall periodontal ligament area available for support and facilitated favorable distribution of functional loads across the long edentulous span, thereby enhancing prosthesis stability and resistance to rotational forces. The definitive restoration consisted of an all-ceramic fixed dental prosthesis fabricated using a customized incisal guide table constructed with the admix technique to accurately reproduce patient-specific mandibular movements and establish harmonious occlusion.

Administration of Therapeutic Intervention

Phase I - diagnostic evaluation and wax-up phase: The treatment procedure was explained in detail to the patient, and written informed consent was obtained for the proposed treatment. Following comprehensive clinical and radiographic examination, diagnostic impressions of the maxillary and mandibular arches were made using irreversible hydrocolloid impression material. The resulting casts were poured in dental stone and mounted on a semi-adjustable articulator using a facebow transfer and centric relation record. Occlusal analysis revealed loss of anterior guidance due to the absence of mandibular incisors.

A diagnostic wax-up was performed to establish the proposed tooth position, contour, esthetics, and functional occlusal scheme. Particular emphasis was placed on developing appropriate anterior guidance to facilitate posterior disocclusion during protrusive and lateral mandibular movements. The diagnostic wax-up served as a blueprint for the subsequent provisional and definitive restorations and allowed evaluation of the anticipated esthetic and functional outcomes before initiation of definitive treatment.

To transfer the established anterior guidance accurately to the semi-adjustable articulator, a customized incisal guide table was fabricated using the admix technique. The incisal guide table of the Hanau Wide-Vue articulator was customized using an admix material consisting of impression compound and greenstick compound in a 3:1 ratio by weight. The mixture was softened in a water bath and adapted onto the mechanical incisal guide table of the articulator. The diagnostic wax-up was then used to guide protrusive and lateral movements of the articulator while the material remained moldable (Figure 3). These movements generated customized pathways corresponding to the patient's anterior guidance. After cooling and hardening, the customized guide table preserved the functional mandibular pathways and allowed their reproduction throughout the laboratory procedures.

**Figure 3:**
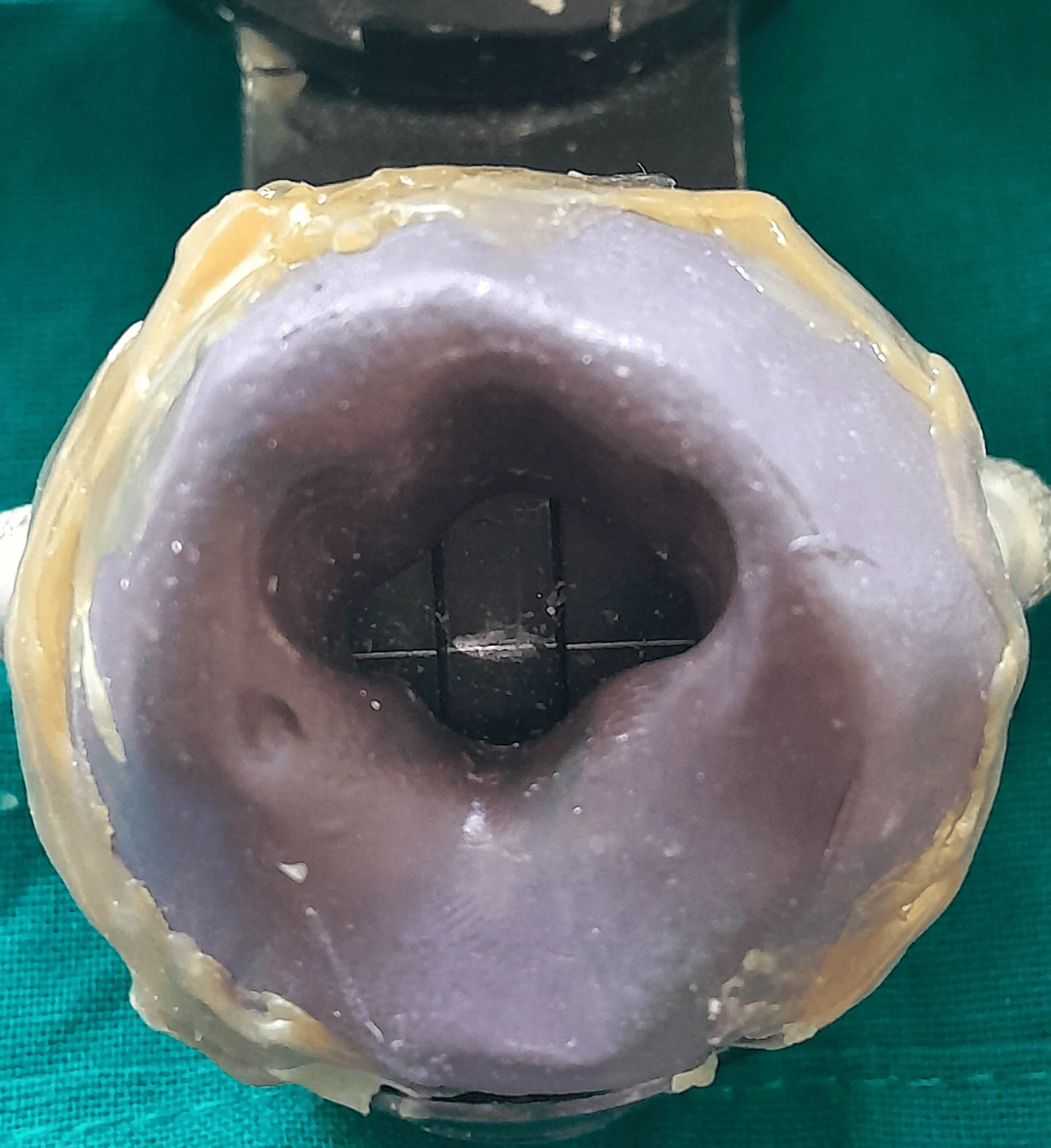
Fabrication of the customized incisal guide table using the admix technique to record patient-specific protrusive and lateral mandibular pathways

Phase II - provisional restoration phase: After completion of the diagnostic phase, the selected abutment teeth (33, 34 and 43, 44) were prepared. Double abutments were selected to increase the periodontal supporting area, improve compliance with Ante's principle, enhance resistance to torsional and bending stresses associated with a long-span prosthesis, and provide stable support for the re-established anterior guidance and canine-protected occlusal scheme. Adequate tooth reduction was achieved to provide sufficient restorative space while maintaining tooth structure and periodontal health. A provisional fixed dental prosthesis was fabricated (Figure 4) using heat-cure tooth-colored polymethylmethacrylate material based on the diagnostic wax-up and cemented using temporary luting cement. The provisional restoration served as a functional prototype for evaluating esthetics, phonetics, comfort, occlusal stability, and anterior guidance.

**Figure 4:**
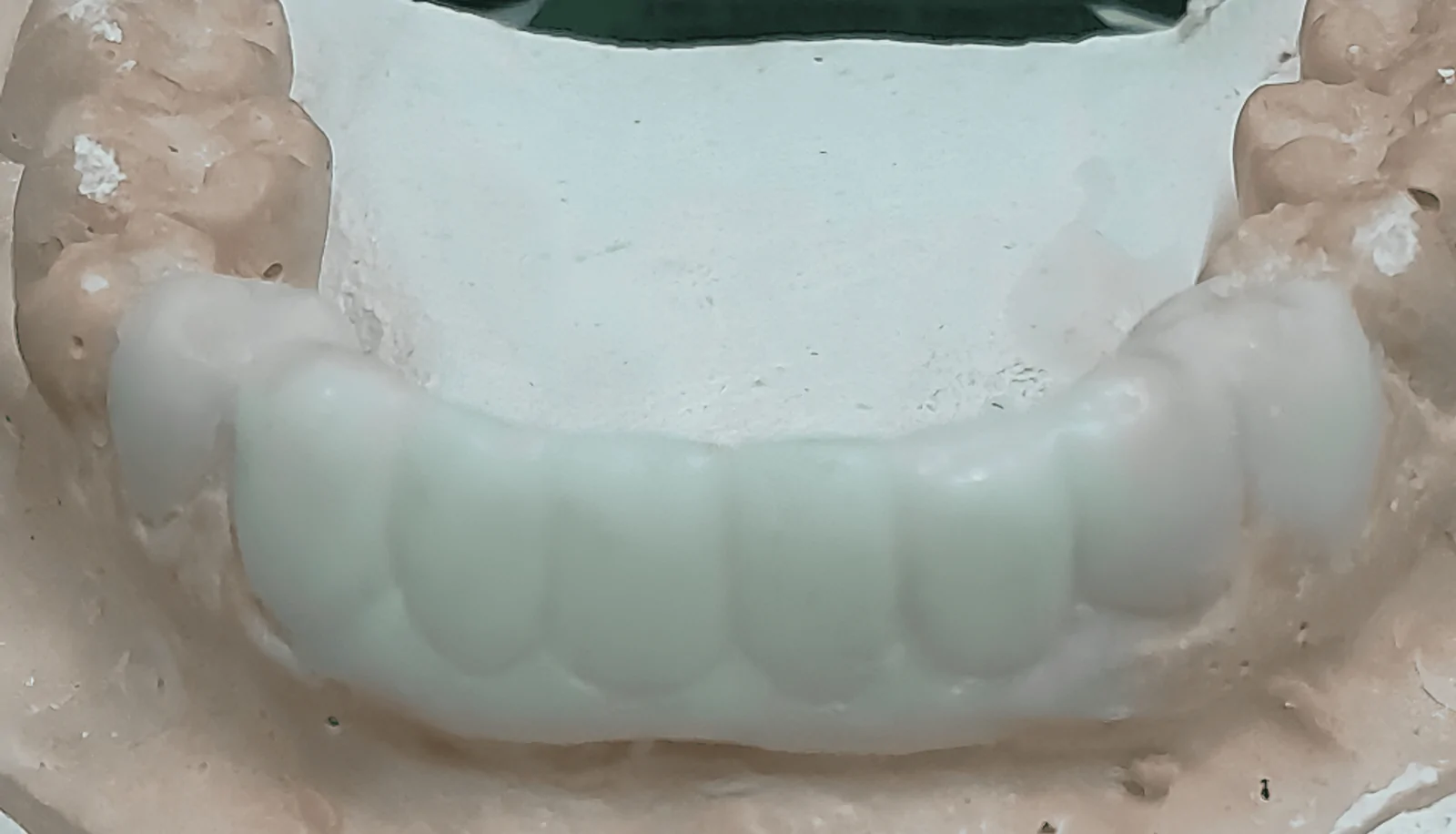
Provisional fixed dental prosthesis fabricated according to the diagnostic wax-up

During this phase, the patient was assessed for adaptation to the proposed occlusal scheme. Protrusive and lateral mandibular movements were carefully evaluated to verify the effectiveness of the established anterior guidance and posterior disocclusion. Necessary occlusal refinements were performed to eliminate interferences and optimize functional movements. The provisional phase also provided an opportunity to assess patient satisfaction with the esthetic outcome and to confirm the suitability of the proposed treatment plan before fabrication of the definitive prosthesis.

The customized incisal guide table was further refined, if necessary, based on the functional movements observed with the provisional restoration. This ensured accurate transfer of the verified anterior guidance to the definitive prosthesis.

Phase III - definitive prosthesis phase: Following successful evaluation of the provisional restoration, definitive impressions were made using an elastomeric impression material. Master casts were fabricated and mounted on the semi-adjustable articulator using the previously established records and customized incisal guide table.

The definitive all-ceramic fixed dental prosthesis was fabricated utilizing the customized guide table to reproduce the verified anterior guidance and functional mandibular pathways established during the provisional phase. This allowed accurate replication of protrusive and lateral movements during laboratory procedures and minimized the need for extensive intraoral occlusal adjustments.

The patient exhibited a bilateral canine-guided, mutually protected occlusal scheme. During protrusive movement, anterior guidance was provided by the maxillary and mandibular anterior teeth, resulting in complete bilateral posterior disocclusion. During right and left lateral excursions, guidance was provided exclusively by the respective working-side canine, with complete disocclusion of the posterior teeth on both the working and non-working sides. Final occlusal adjustments were performed to establish harmonious centric contacts and consistent anterior guidance during eccentric mandibular movements, resulting in posterior disocclusion during protrusive (Figure 5), right lateral (Figure 6), and left lateral (Figure 7) excursions.

**Figure 5:**
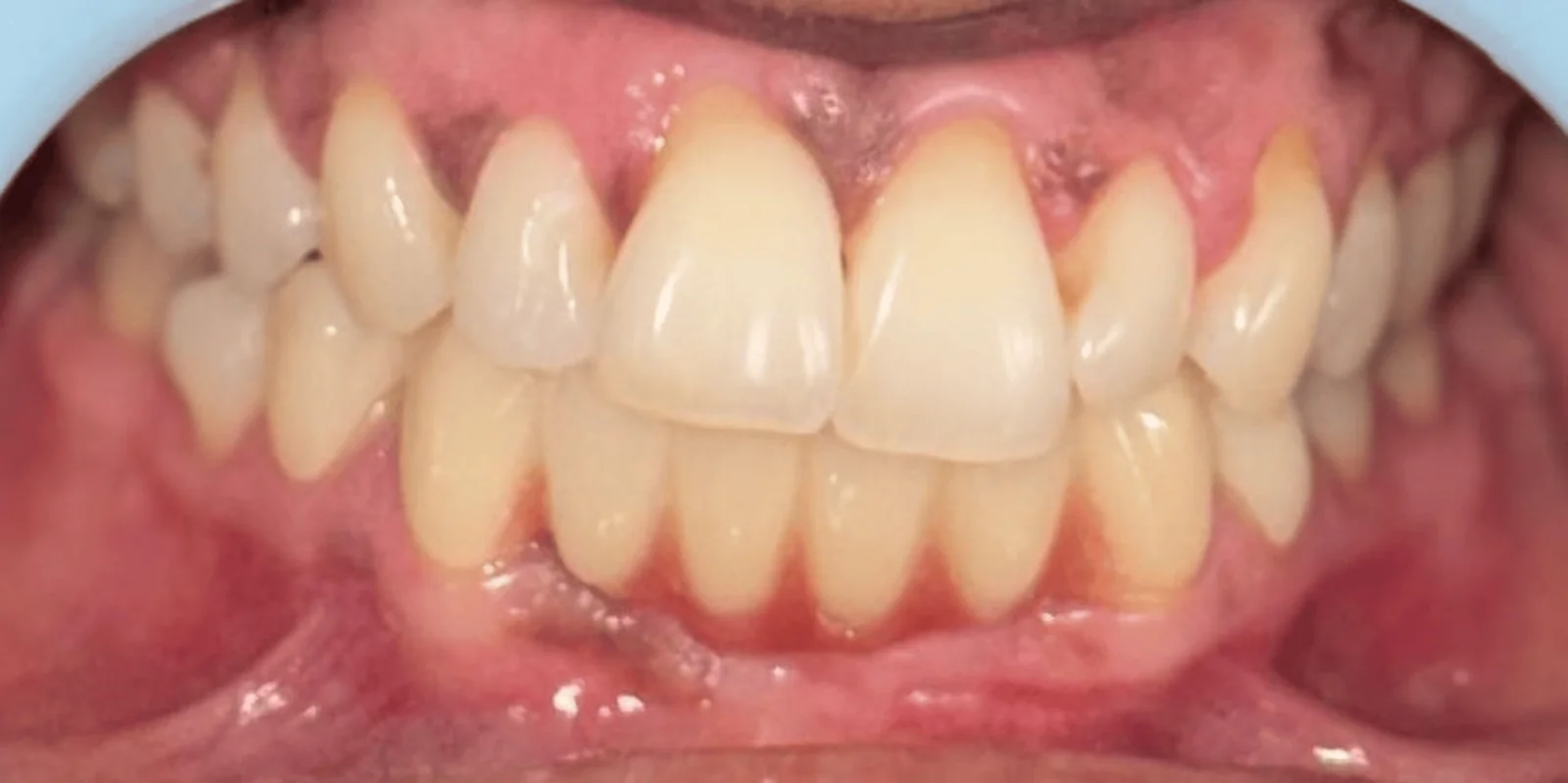
Clinical evaluation of the definitive prosthesis demonstrating centric occlusion

**Figure 6:**
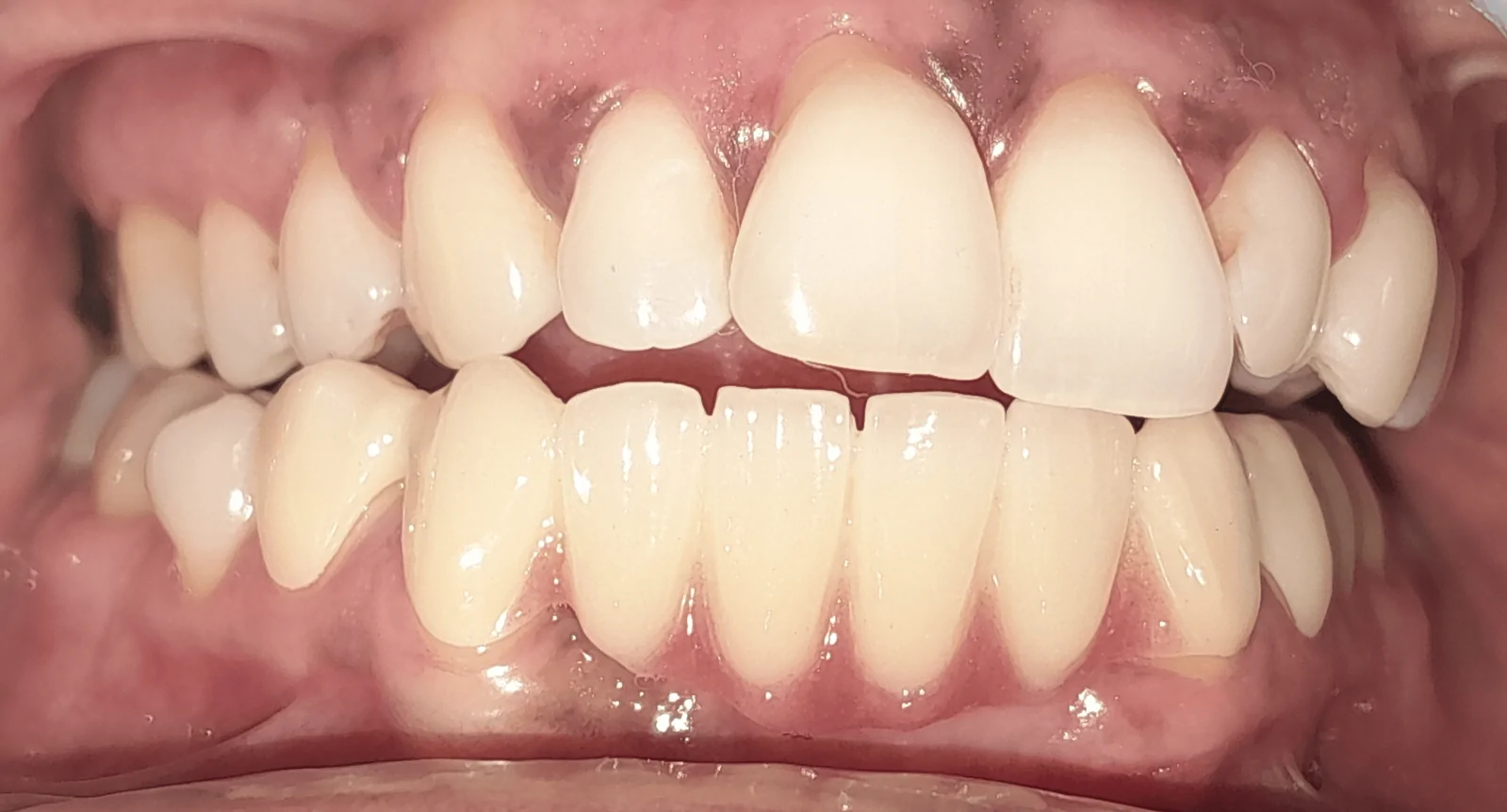
Occlusion at the right lateral excursion with canine guidance and posterior disocclusion

**Figure 7:**
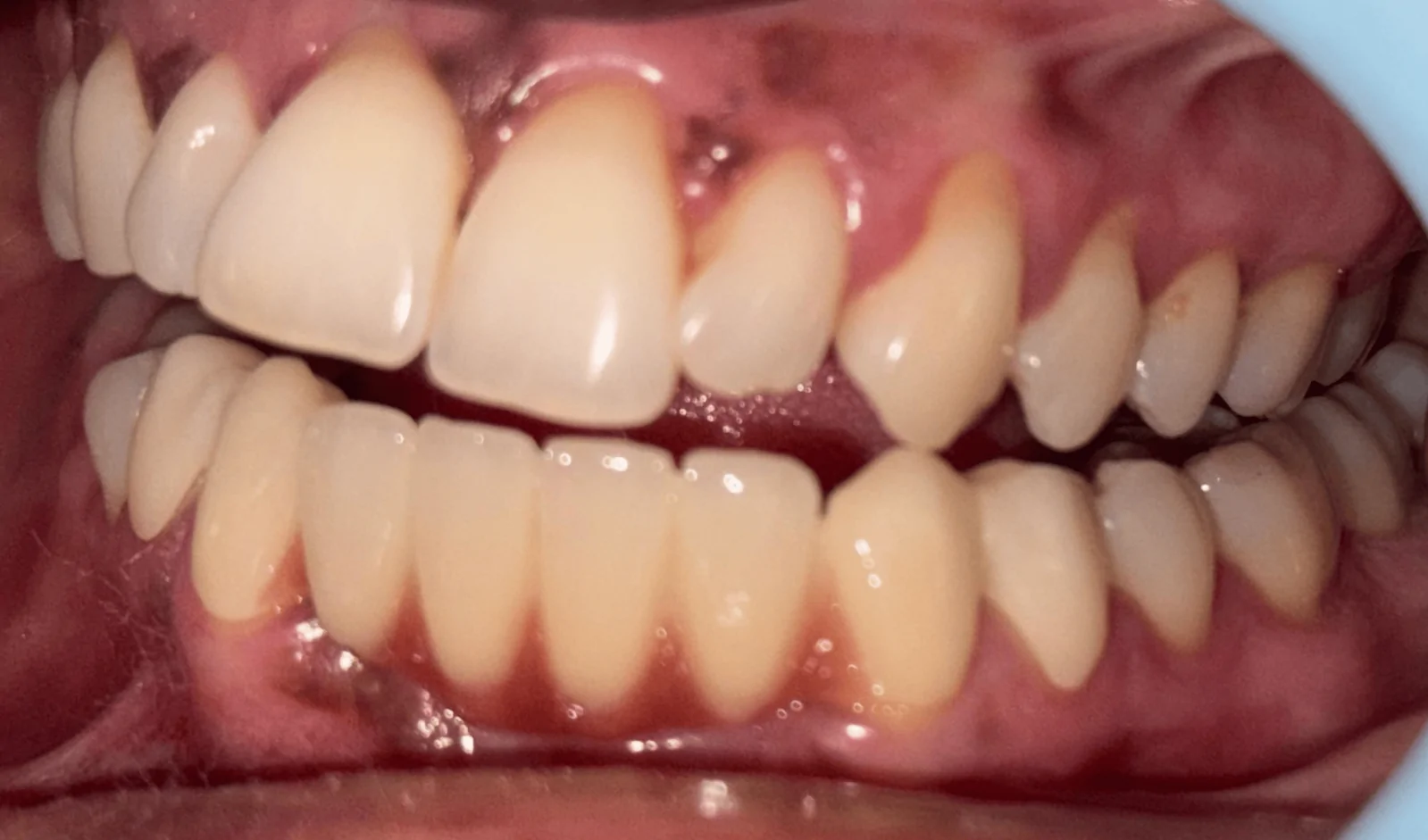
Occlusion at the left lateral excursion with canine guidance and posterior disocclusion

At the cementation appointment, the definitive prosthesis was evaluated for marginal adaptation, proximal contacts, esthetics, phonetics, and occlusal relationships. Following confirmation of satisfactory fit and function, the prosthesis was cemented.

Postoperative instructions regarding oral hygiene maintenance and prosthesis care were provided. The patient was recalled periodically for follow-up evaluation. At the six-month review, the prosthesis demonstrated satisfactory esthetic and functional performance, stable anterior guidance, effective posterior disocclusion, and absence of biological or mechanical complications.

Changes in Therapeutic Intervention

No modifications to the planned treatment protocol were required during treatment. The customized incisal guide table fabricated using the admix technique provided satisfactory transfer of functional mandibular movements and facilitated fabrication of the definitive prosthesis without the need for alteration of the treatment plan.

Follow-up and outcomes

Clinician- and Patient-Assessed Outcomes

At the six-month follow-up visit, the patient reported improvement in masticatory efficiency, comfort during function, speech, and overall satisfaction with the esthetic outcome of the treatment. Clinical evaluation revealed stable anterior guidance, harmonious occlusion, and effective posterior disocclusion during protrusive and lateral mandibular movements. No occlusal interferences or functional limitations were observed.

Follow-up

Periodic clinical examinations demonstrated healthy periodontal tissues surrounding the abutment teeth and satisfactory function of the fixed prosthesis. The prosthesis remained stable and intact, with no evidence of debonding, fracture, or wear. Radiographic evaluation using an orthopantomogram (Figure 8) at the six-month follow-up revealed no pathological changes involving the abutment teeth or supporting structures. The endodontically treated abutment teeth remained asymptomatic, and no radiographic signs of periapical pathology or periodontal deterioration were observed.

**Figure 8:**
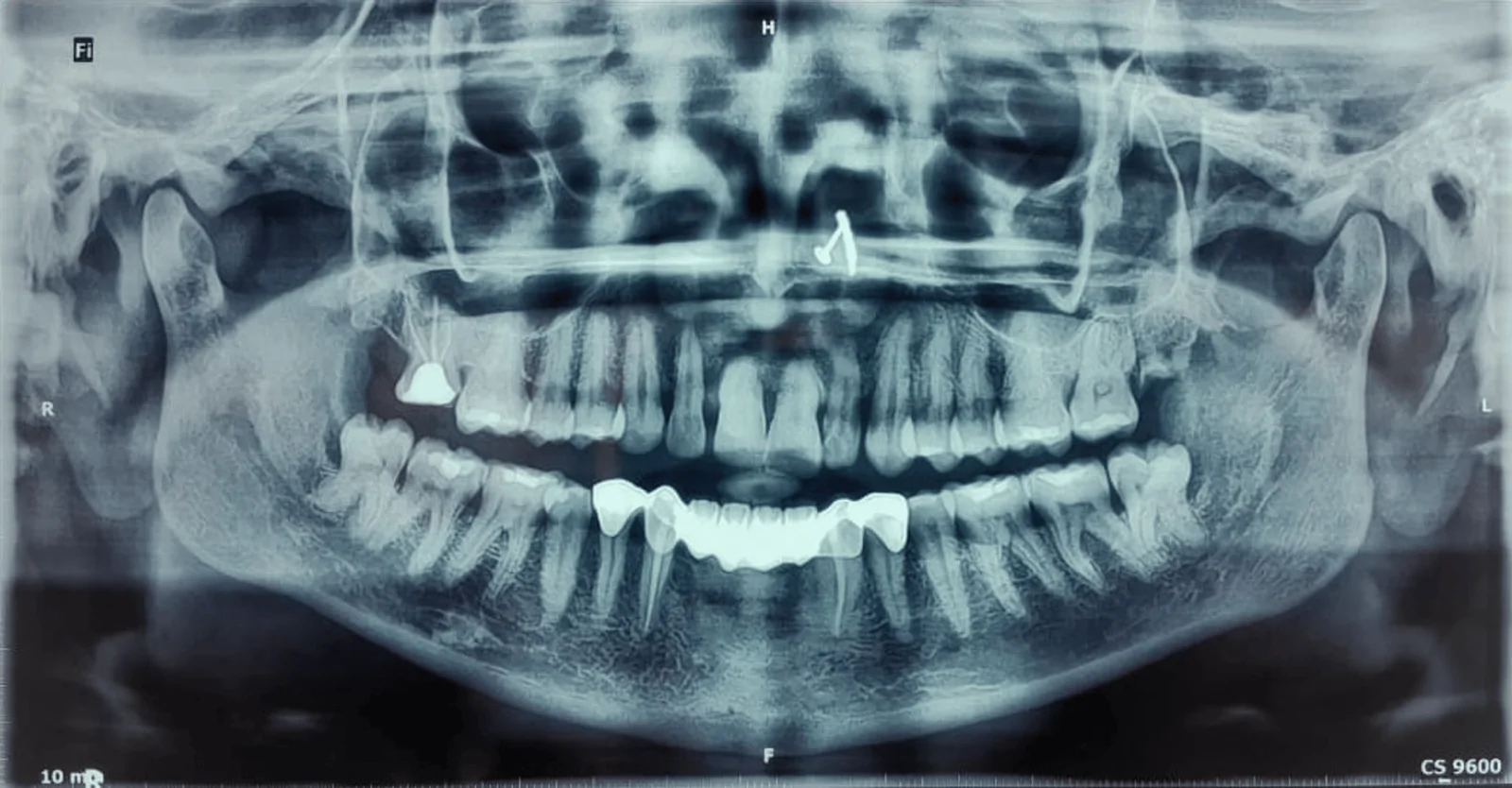
Six-month postoperative orthopantomogram showing satisfactory definitive prosthesis and supporting structures

Intervention Adherence and Tolerability

Adherence to the treatment protocol was assessed through attendance at scheduled follow-up appointments and evaluation of oral hygiene maintenance. The patient complied with postoperative instructions and maintained satisfactory oral hygiene throughout the follow-up period. The prosthesis was well tolerated, and no difficulties related to mastication, speech, comfort, or adaptation were reported.

Adverse and Unanticipated Events

No biological, mechanical, or prosthetic complications were observed during the six-month follow-up period. No adverse or unanticipated events were reported.

## Discussion

Strengths and limitations

The main strength of this approach was the use of a customized incisal guide table fabricated with admix material to facilitate transfer of patient-specific mandibular movements to a semi-adjustable articulator. Its thermoplastic nature allowed easy modification and refinement of protrusive and lateral pathways during the diagnostic and provisional stages. The technique was simple, economical, and readily available.

However, this single case cannot establish the superiority of admix material over other materials. The accuracy and dimensional stability of the guide table were not objectively measured, the procedure was operator-dependent, and the six-month follow-up limits assessment of long-term performance.

Discussion of relevant literature

Anterior guidance plays an important role in directing eccentric mandibular movements and facilitating posterior disocclusion [2-4]. Customized incisal guide tables have been described as a means of reproducing individualized anterior guidance during restorative treatment [5,8-10]. Different materials, including light-polymerized provisional materials and vinyl polysiloxane, have subsequently been used for this purpose [11,12]. The transfer of anterior guidance established during provisionalization to the definitive restoration has also been described [13]. A clinical case report by Mall et al. further demonstrated the application of a customized incisal guide table during anterior rehabilitation [8]. More recently, digital workflows have been reported for transferring anterior guidance from provisional to definitive restorations [14]. The present technique follows the same fundamental principle using a conventional, simple, and modifiable admix material.

## Conclusions

The conclusions are based on the successful clinical application of the customized guide table and the six-month clinical outcome. The technique facilitated transfer of the established anterior guidance to the definitive prosthesis, resulting in satisfactory occlusion, esthetics, and function without observed complications. However, these findings demonstrate clinical feasibility rather than efficacy or superiority. Further comparative studies are required to evaluate the accuracy, reproducibility, and dimensional stability of admix material.

This case demonstrates that an admix-material customized incisal guide table can provide a simple, economical, and modifiable method for transferring patient-specific anterior guidance during fixed prosthodontic rehabilitation. When combined with careful clinical recording, provisional evaluation, and occlusal verification, the technique can facilitate harmonious occlusion and satisfactory functional and esthetic outcomes.
